# Texas State Medical Association

**Published:** 1899-02

**Authors:** Joseph Mullen, R. E. Moss

**Affiliations:** Chairman; Houston, Texas; Secretary; Houston, Texas


### Society Notes.

### Texas State Medical Association.

HOUSTON, TEXAS, January 30, 1899.

MY DEAR DOCTOR: Your chairman of the Section on the Eye, Ear, Nose and Throat desires, with your co-operation, to have the work of this section at the San Antonio meeting of the most practical and clinical in character, and to this end he earnestly requests your contribution on any diseased condition or therapeutic observation in which you are interested on the eye, ear, nose and throat.

With your experience in special or general medicine you can, and it is to be hoped will give the meeting the benefit of that experience, which he feels sure will be appreciated by the Association, and especially by himself.

He trusts also to have the pleasure of meeting you in San Antonio, April 25th, 26th, 27th and 28th.

Cordially yours,

JOSEPH MULLEN, Chairman.

R. E. MOSS, Secretary.

P. S.—Subject assigned is "Treatment of Granulated Lids." You are earnestly requested to participate in the discussion of this very *practical* subject.

